# Role of Hepcidin in the Setting of Hypoferremia during Acute Inflammation

**DOI:** 10.1371/journal.pone.0061050

**Published:** 2013-04-23

**Authors:** Jean-Christophe Deschemin, Sophie Vaulont

**Affiliations:** 1 Institut National de la Santé et de la Recherche Médicale, U1016, Institut Cochin, Faculté de Médecine Cochin Port Royal, Paris, France; 2 Centre National de la Recherche Scientifique, UMR8104, Paris, France; 3 Université Paris Descartes, Sorbonne Paris Cité, Paris, France; National Institute of Child Health and Human Development, United States of America

## Abstract

The anemia of chronic disease (also called anemia of inflammation) is an acquired disorder of iron homeostasis associated with infection, malignancy, organ failure, trauma, or other causes of inflammation. It is now widely accepted that induction of hepcidin expression in response to inflammation might explain the characteristic hypoferremia associated with this condition. To determine the role of hepcidin in acute inflammation and the regulation of its receptor, the iron exporter, ferroportin, wild-type, heterozygote and hepcidin knockout mice (Hepc−/−) were challenged with sublethal doses of lipopolysaccharide (LPS). Six hours after injection, ferroportin mRNA and protein levels were assessed in the duodenum and the spleen and plasma iron was determined. Our results demonstrate that hepcidin is crucial, though not the sole mediator of LPS-mediated acute hypoferremia, and also that hepcidin major contribution relies on decreased ferroportin protein levels found in the spleen. Furthermore, we establish that LPS-mediated repression of the membrane iron transporter DMT1 and oxidoreductase Dcytb in the duodenum is independent of hepcidin. Finally, our results in the hepc+/− mice indicate that elevated hepcidin gene expression is not a prerequisite for the setting of hypoferremia during early inflammatory response, and they highlight the intimate crosstalk between inflammatory and iron-responsive pathways for the control of hepcidin.

## Introduction

One of the most potent pathogen-derived inflammatory signals is the Gram-negative bacterial cell wall component lipopolysaccharide, LPS, which induces a massive release of cytokines and other inflammatory mediators in the infected host. This release is critical for the integration of the innate immune response, but requires tight regulation to prevent subsequent inflammation, which may lead to endotoxic shock, a condition that is still one of the most frequent causes of mortality in the hospital intensive care unit. It is now well established that LPS mediates its effects through a member of the highly conserved Toll-like receptor (TLR) family, TLR4, for the induction of proinflammatory genes [1]. Endotoxic shock is not only associated with an extreme proinflammatory response but is also accompanied by hypoferremia, a primitive defensive mechanism which contributes to allowing reducing circulating iron, while minimising its availability to pathogens (for review, [2]). Hepcidin has emerged as the possible pathogenic mediator of hypoferremia. Hepcidin functions as the master regulator of cellular iron export by controlling the amount of cell surface ferroportin, an iron exporter which is present predominantly on the basolateral surface of intestinal enterocytes and macrophages. Hepcidin binding to ferroportin induces its internalization and degradation, resulting in cellular iron retention, decreased iron export and hypoferremia (for review, [3]). While hepcidin was found up-regulated by LPS in several species (referenced in [4]), from mice [5] to humans [6], the impact of this increase in the acute setting of inflammation-induced hypoferremia remains to be established. Here, we take advantage of the hepcidin knockout mouse model [7] to address this question by using the well-described model of LPS injection.

## Materials and Methods

### Animals

#### Ethic statement

Mice were cared for in accordance with the European convention for the protection of laboratory animals. Animal studies received approval from the Regional Ethics Committee for Animal Experimentation of University Paris Descartes. Animals were given free access to tap water and a standard laboratory mouse chow diet (AO3, iron content 280 mg/kg, UAR, France). Age-matched wild-type (WT), heterozygote (Hepc +/−) and homozygote (Hepc−/−) male mice of 8-13-week-old on a C57BL/6 background were used in this study. Plasma iron was determined as previously described [7].

Inflammation was induced by intra-peritoneal injection of LPS (from *E.coli* O111:B4; 2 mg/kg). Control mice were injected with sterile saline solution. Mice were sacrificed 6 hours later. Results are presented for a series of n = 3 animals per group and has been reproduced at least twice on different set of animals.

### RNA and protein analysis

RNA extraction and real-time quantification of transcripts were performed as described [8]. mRNA expression was calculated using the ΔΔCt method and normalized to the expression of cyclophilin. Primers used are outlined in Table S1.

Spleen microsomal and cytosolic fractions were prepared as previously [9]. Briefly, spleens were ground with tungsten beads in a TissueLyser in histidine/sucrose buffer with EDTA-free protease inhibitor cocktail. The lysate was centrifuged at 5000 rpm for 10 min to eliminate nuclei and unbroken cells. The protein extracts were then ultracentrifuged at 42000 rpm for 1hr at 4°C to separate the crude membrane fractions from the cytosolic proteins. Enterocytes from duodenum were enriched as follows. Proximal duodenum was placed in a 1.5mM EDTA PBS supplemented with PMSF and protease inhibitors and left to shake for 45 min at 4°C. The remaining duodenal muscle was removed and detached enterocytes spun down. The cells were treated with lysis buffer (0.25M sucrose, 0.03M L-Histidine, pH = 7.2, 500μM PMSF and protease inhibitors) for 30 min. The samples were then spun down, the supernatant was removed and spun at 42000 rpm for 1hr to obtain the cytosolic fraction (supernatant) and membrane fraction (pellet).

Anti-ferroportin and anti-Dcytb antibodies were from Alpha Diagnostics, anti-βactin antibodies from Sigma and anti-lipocalin-2 antibodies from R&D Systems. Anti-DMT1 were kindly provided by F Canonne-Hergaux [10], [11]. Proteins were visualized using Image Quant Las4000 mini (GE Healthcare) and quantified with ImageJ.

### Statistical analysis

Statistical analysis was performed using Student *t* test (unpaired, 2 tailed). *P* values less than .05 were considered statistically significant.

## Results and Discussion

### Adequate inflammatory response to LPS in Hepc**−/−** mice

To study the LPS response in Hepc−/− mice, we first determined whether the acute phase response to LPS was altered in these mutant mice. For that, we measured the mRNA levels of the pro-inflammatory cytokines Tumor necrosis factor-α (TNF-α) and Interleukin-6 (IL6) that are induced in response to LPS to trigger the expression of acute phase genes. Regardless the genotype, expression of TNF-α and IL6 was dramatically increased in response to LPS in both liver and spleen (Figure S1). Similarly, mRNA levels of Heme oxygenase-1, a well-known modulator of inflammation, were also enhanced in the liver of LPS treated WT and Hepc−/− mice (Figure S1). Finally, mRNA levels of activin B, a member of the TGF-beta superfamily that was recently described as crucial for hepcidin induction by inflammation [12] was examined and found up-regulated by LPS in both WT and Hepc−/− mice (Figure S1). Overall, these results indicate that LPS is equally efficient at mounting an inflammatory response in WT and Hepc−/− mice. As expected, in WT mice, LPS injection induced an increase of hepcidin mRNA level in the liver of about 2 fold (Figure S2).

### LPS treatment of Hepc**−/−** mice results in a dramatic decrease of ferroportin protein levels in the duodenum but in not the spleen

**Figure 1:**
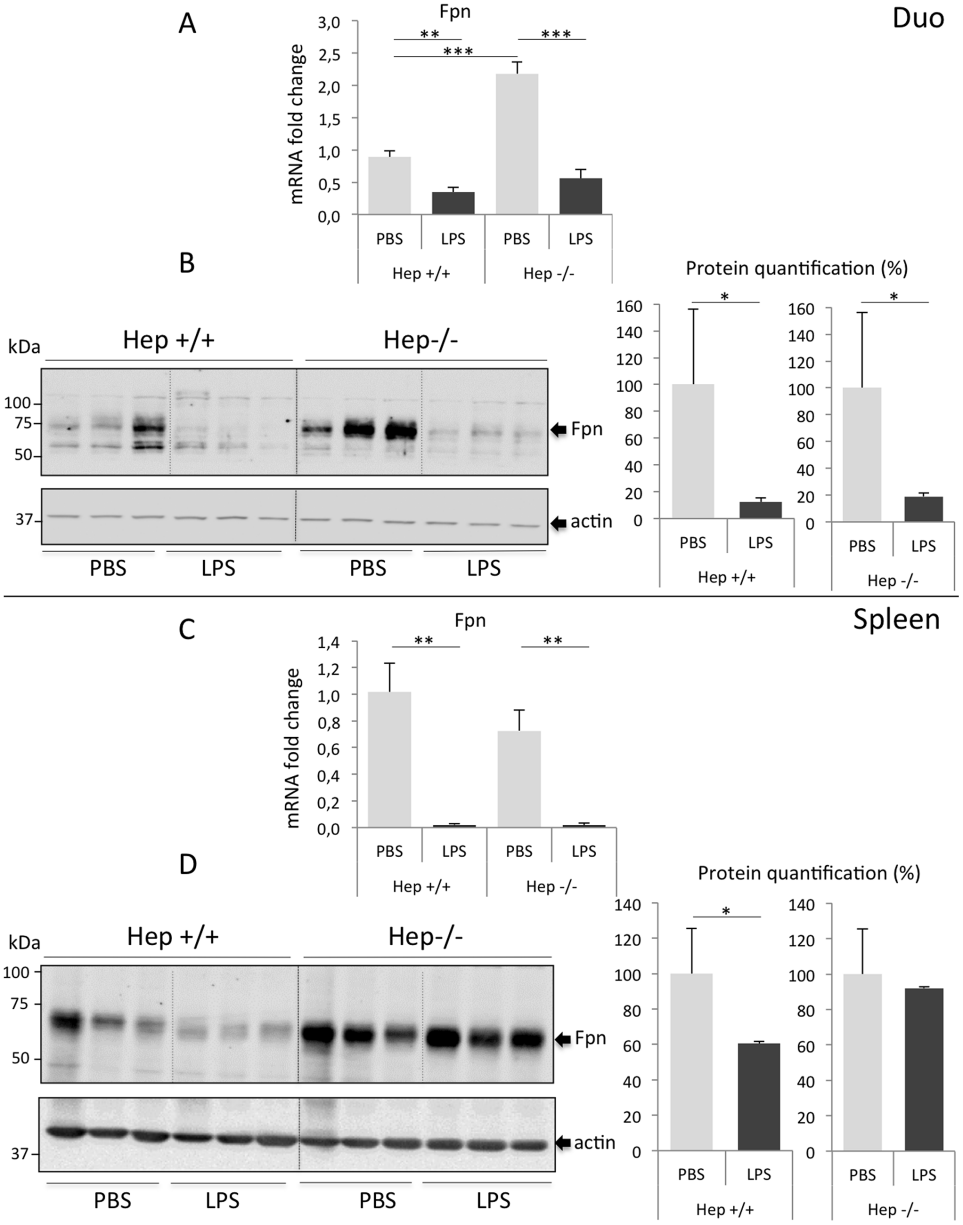
Ferroportin expression in duodenum, and spleen of WT and Hepc−/− mice treated with PBS or LPS. Relative changes in ferroportin mRNA levels were quantified by real-time qRT-PCR. Graphed are mean values that were obtained from mRNA expression analyses regarding cyclophilin in the duodenum (duo), top panel, and the spleen, lower panel. Results are expressed relative to control PBS treated WT mice. Western Blot of ferroportin on membrane extracts were performed with 5 µg/lane for the duodenum and 20 µg/lane for the spleen. Expression was normalized to beta actin and quantified using Image J. Quantification of the blots are presented in % (100% set for Hepc+/+ and Hepc−/− mice, respectively). Error bars represent SD for n = 3 mice in each group. Statistical significance is indicated by * symbols (**p< 0.005, ***p< 0.0005).

The effect of LPS on ferroportin level was analyzed in the two critical sites contributing to circulating iron, the duodenum (site of dietary iron absorption) and the spleen (containing tissue macrophages dedicated to iron recycling from hemoglobin catabolism). After 6h of LPS treatment, ferroportin mRNA levels were found decreased in both tissues, regardless the genotype (Figure 1). In the duodenum, while mRNA decreased 2- and 4-fold in WT and Hepc−/− mice, respectively (Figure 1A), ferroportin decrease was much more pronounced at the protein levels, in both WT and mutant mice (almost 90%, Figure 1B), suggesting the involvement of additional hepcidin-independent process to down-regulate ferroportin protein levels in this tissue during acute inflammation. Interestingly, we observed some differences in the regulation of ferroportin in response to LPS in the spleen when compared to the duodenum. In the spleen, decreased ferroportin mRNA levels by LPS was much more dramatic, 50-and 36-fold, in WT and Hepc−/− mice, respectively (Figure 1C). However, in WT mice, only a moderate but significant 40% reduction of ferroportin protein level was observed. In addition, in Hepc−/− mice, in contrast to the duodenum where ferroportin mRNA decrease was accompanied by protein decrease, ferroportin protein level was not altered by LPS treatment (Figure 1D). Notably, and as previously reported in hepcidin deficient mice [9], basal ferroportin protein level was found up-regulated in the mutant duodenum and spleen due to the lack of hepcidin. Together, these results confirm previous studies demonstrating ferroportin inhibition by LPS [13], [14] and further indicate that LPS-mediated transcriptional repression or selected degradation of RNA of ferroportin is independent of hepcidin both in the duodenum and in the spleen. Noteworthy, the same inhibitory effect of LPS was obtained for the two recently reported isoforms of ferroportin 1A and 1B in the duodenum and in the spleen [15] (Figure S3). TLR4 was reported to be critical for LPS-mediated down-regulation of ferroportin in the spleen [13], independently of MyD88, as recently suggested [16]. Whether TLR4/MyD88/TRIF signaling is required in the duodenum remains to be determined.

Second, our results establish that in the duodenum, ferroportin protein levels seem highly sensitive to acute inflammation. In this tissue, hepcidin deficiency is unable to counteract the inflammatory signals, i.e. to upregulate ferroportin levels, suggesting that the inflammatory signals are dominant over the lack of hepcidin in the regulation of ferroportin levels. In contrast, the amount of membrane ferroportin protein in the spleen is dependent on hepcidin in the setting of acute inflammation, hepcidin deficiency leading to reduce ferroportin degradation as compared to WT mice (i.e. acute suppression of ferroportin protein by LPS being efficient only in WT mice and not in Hepc−/− mice).

### LPS-induce hypoferremia relies on both hepcidin-dependent and -independent mechanisms

**Figure 2:**
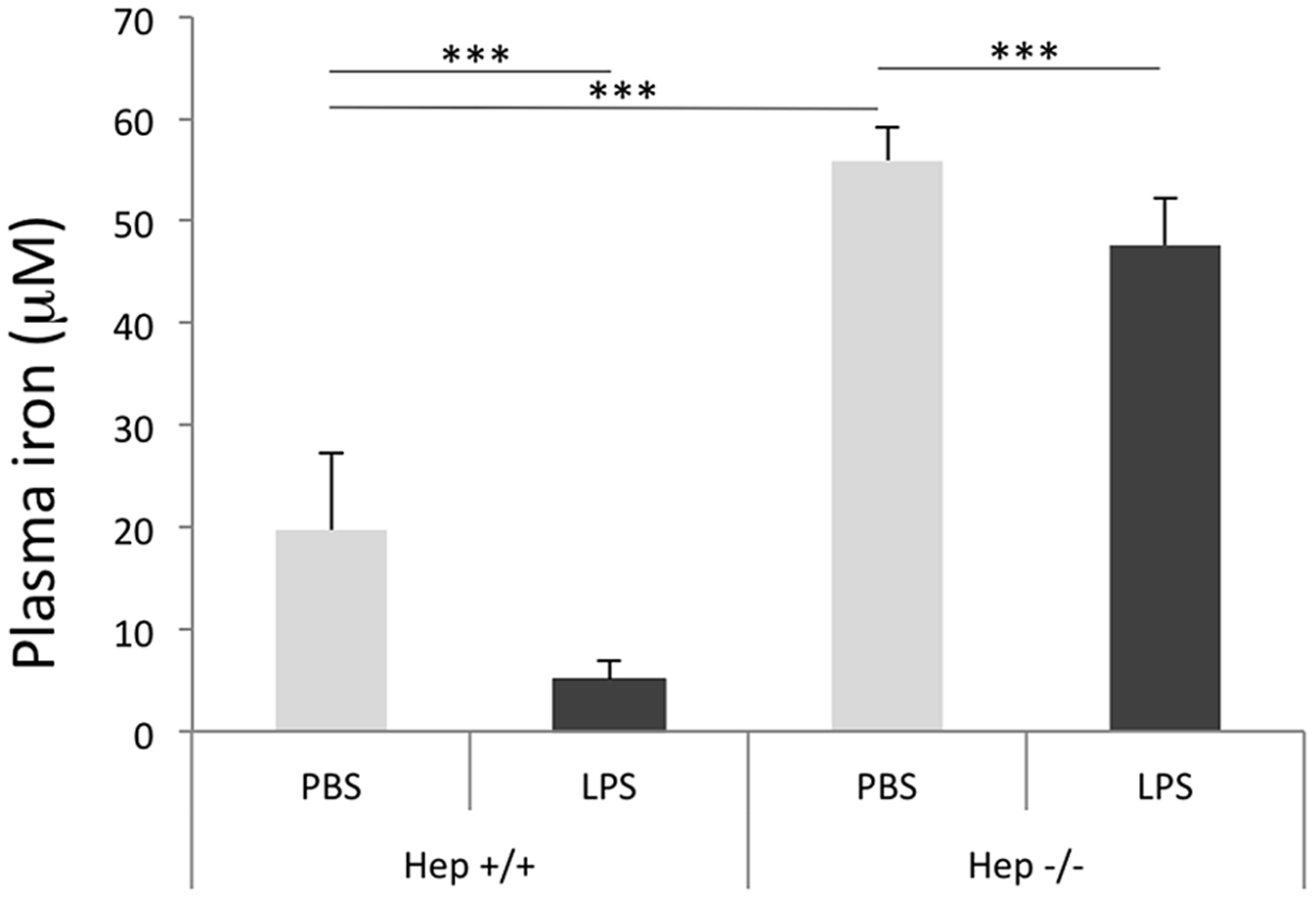
Plasma iron in WT and Hepc−/− mice treated with PBS or LPS. Error bars represent SD for n = 3 mice in each group. Statistical significance is indicated by * symbols (***p< 0.0005).

The next step was to evaluate the physiological consequence of hepcidin/ferroportin changes after LPS treatment in plasma iron content. The moderate (spleen) and drastic reduction of ferroportin (duodenum) proteins in the WT treated mice yielded a 75% decrease of plasma iron (Figure 2). In the Hepc−/− mice, as expected [9], plasma iron level was signicantly high with regards to WT mice. In these mutant mice, LPS-mediated hypoferremia was largely blunted, although duodenum ferrorportin levels were similarly reduced in comparison with WT treated mice. This result suggests that ferroportin regulation in the spleen impacts more on plasma iron concentrations than ferroportin regulation in the duodenum. Interestingly, abrogation of hypoferremia in Hepc−/− mice was not complete, LPS still inducing a significant 15% decrease of plasma iron (Figure 2). This result clearly indicates the involvement of other signals independent of hepcidin contributing to the rapid hypoferremia. Since lipocalin-2 (Lcn2), a peptide involved in bacterial iron sequestration, was recently described to play a key role in hypoferremia of inflammation in a model of LPS-induced sepsis [17], we asked whether a decrease in Lcn2 in the Hepc−/− would contribute to the reduction of hypoferremia as observed in these mice. Although we confirmed that Lcn2 gene expression was dramatically increased in the liver of WT LPS treated animals, both at mRNA and protein levels, we found similar activation of Lcn2 (or even higher activation) in Hepc−/− treated mice (Figure S4).

### Similar regulation by LPS of iron-related gene expression in WT and Hepc**−/−** mice

**Figure 3:**
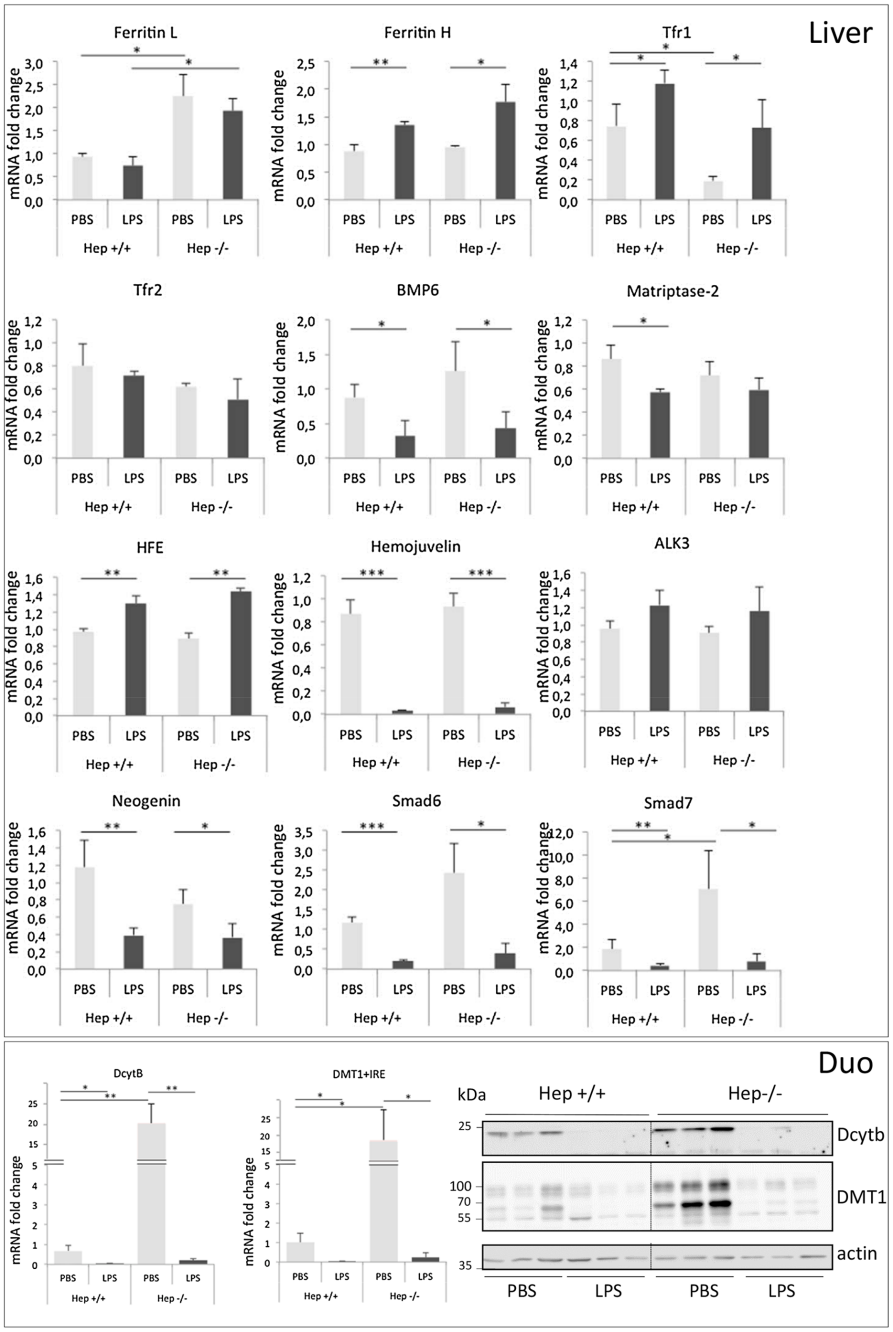
Relative mRNA levels and proteins of indicated genes in the liver and the duodenum of WT and Hepc−/− mice treated with PBS or LPS. Relative changes in mRNA levels were quantified by real-time qRT-PCR in the liver, top panel and the duodenum (duo), low panel. Western Blot of Dcytb and DMT1 on membrane extracts were performed with 25 µg/lane. Error bars represent SD for n = 3 mice in each group. Statistical significance is indicated by * symbols (*p< 0.05, **p< 0.005, ***p< 0.0005).

A close examination of mRNA levels of liver iron-related gene expression was performed. In brief, genes that were either unaffected (ferritin L, Tfr2, Alk3), increased (ferritin H, Tfr1, HFE), or decreased (BMP6, matriptase-2, hemojuvelin, neogenin, Smad6 and Smad7) by LPS treatment in WT mice followed exactly the same trend in Hepc−/− mice (Figure 3, upper panel), indicating that hepcidin was not required for these LPS-mediated regulations. Interestingly, we found that, in the duodenum, LPS triggered a decrease not only of ferroportin mRNA and protein levels but also of the apical membrane proteins Dcytb, Duodenal cytochrome *b*, and DMT1, divalent metal transporter 1 (both at mRNA and protein levels, Figure 3, lower pannel), thus confirming the general strategy of the body to decrease dietary iron uptake during inflammation. However, in contrast to previous assumptions [18], [19], our results permit to exclude a role of hepcidin in the short-term regulation of these iron-related proteins by inflammation (since they were similarly down regulated in the Hepc−/− mice, Figure 3), and further support a role of the inflammatory cytokines for the rapid repression of ferroportin, Dcytb and DMT1 mRNA levels in the duodenum.

### LPS response in Hepc**+/−** mice: the hepatic iron load may impact on LPS-mediated hepcidin induction

In this study, LPS was also injected in heterozygote Hepc+/− mice. Surprisingly, hepcidin gene expression was not increased in the liver of these mice (Figure S5) although LPS injection triggered similar responses as compared to WT mice, i.e., a 75% reduction of plasma iron (Figure S5) and similar changes in gene expression (not shown). This result is reminiscent to the detected hypoferremia despite the lack of hepcidin induction in other mouse models of inflammation such as mycobacteria-infected mice [20]; LPS treated TRIF-deficient mice [16]; TNF-α-injected mice [21]; and a model of peritonitis induced by cecal ligation puncture [22]. Together, these results establish that elevated hepcidin gene expression is not a prerequisite for the setting of hypoferremia during the early inflammatory response and suggest that hepcidin is necessary but that it is not the sole mediator for the development of hypoferremia.

Why hepcidin does not increase in heterozygote Hepc+/− mice after LPS injection is not yet clear. One explanation might be the mild but highly significant 1.5-fold increase in liver iron measured in these heterozygote mice (85 ±13 µg/g wet tissue in WT mice n = 11, vs 132 ±22 µg/g wet tissue in Hepc+/− mice, n = 11, p = 2.8E-05). Indeed, recent evidence suggested that the amount of cellular hepatic iron could influence the response of the hepcidin gene to LPS. Villarroel *et al.* demonstrated that LPS-induced hepcidin gene expression in HepG2 cells was completely blunted when the cells were preloaded with holo-Tf or Fe-NTA [23]. Conversely, Pagani *et al.* reported that the fold increase of LPS-mediated hepcidin expression was significantly higher in iron deficient animals as compared to controls [24]. This altered hepcidin response in regards to iron levels is most likely due to the activity of the signaling BMP6/HJV/SMAD pathway (and not iron *per se*). Indeed, in the BMP6 KO mice presenting, similarly to the iron deficient mice [25], a reduced activation of the pathway, the hepcidin response to LPS is greatly enhanced despite the important iron load [26]. Collectively, these results suggest that the BMP/HJV pathway could alter the response of hepcidin to LPS, an assumption that had already been anticipated by Niederkofler *et al.* in 2005 who showed, as reported [27], a rapid and selective extinction of hemojuvelin expression in the liver during acute inflammation [28]. These authors suggested that the iron-sensing pathway had to be switched off during inflammation as to prevent interference and cross-regulatory interactions between individual pathways. This finding is confirmed by the present study showing a significant decrease of hemojuvelin but also of other genes involved in the iron pathways such as BMP6, matriptase-2, neogenin, Smad6 and Smad7. While TNF-α was shown to mediate HJV transcriptional repression by LPS [29], [30], a broader role of this cytokine in this pathway remains to be investigated.

To conclude, our study shows that the development of hypoferremia during acute inflammation involve both hepcidin-dependent and -independent mechanisms. The rapid and coordinate decrease of ferroportin expression (as well as that of other iron-related genes, Figure 3) that is induced by LPS in the major tissues which contributes to body iron delivery occurs regardless of hepcidin. A close examination of the role of endoplasmic reticulum (ER) stress and of how intracellular stress and inflammatory responses interact together for the induction of the acute phase response will deserve further investigations [31]. The major contribution of hepcidin in the immediate developement of hypoferremia relies on decrease ferroportin protein levels in the spleen as ferroportin protein level in the duodenum is shown to have been completely repressed by LPS, even without hepcidin. Furthermore, elevated hepcidin is not necessary for LPS-mediated hypoferremia (as shown in the Hepc+/− mice), thus implying that cooperative mechanisms have to operate to lead to iron redistribution and hypoferremia in this condition. Finally, our results highlight the intimate crosstalk between the inflammatory and iron-responsive pathways in the liver leading to the control of the hepcidin gene.

## Supporting Information

Figure S1 **Relative mRNA levels of indicated genes in liver and spleen of WT and Hepc−/− mice treated with PBS or LPS.** Error bars represent SD for n = 3 mice in each group. Statistical significance is indicated by * symbols (*p< 0.05, **p< 0.005).
(TIFF)

Figure S2 **Relative hepcidin1 mRNA levels in the liver of WT and Hepc−/− mice treated with PBS or LPS.** Error bars represent SD for n = 3 mice in each group. Statistical significance is indicated by * symbols (*p< 0.05).
(TIFF)

Figure S3 **Relative mRNA levels of ferroportin A (FpnA) and ferroportin B (FpnB) in the duodenum and spleen of WT and Hepc−/− mice treated with PBS or LPS.** Error bars represent SD for n = 3 mice in each group. Statistical significance is indicated by * symbols (*p< 0.05, **p< 0.005, ***p< 0.0005).
(TIFF)

Figure S4 **Relative lipocalin-2 (Lcn2) mRNA and protein levels in the liver of WT and Hepc−/− mice treated with PBS or LPS.** rror bars represent SD for n = 3 mice in each group. Statistical significance is indicated by * symbols (**p< 0.005, ***p< 0.0005). For the Western blot, cytosolic proteins (20 µg/lane) were separated on SDS-PAGE, electro-transferred onto nitrocellulose membrane and analyzed with anti-Lcn2 antibody.
(TIFF)

Figure S5 **Relative liver hepcidin1 mRNA levels and plasma iron in Hepc+/− mice treated with PBS or LPS.** rror bars represent SD for n = 3 mice in each group. Statistical significance is indicated by * symbols (***p< 0.0005).
(TIFF)

Table S1 **Primers sequences used for Real-time PCR.**
(DOCX)
