# Supplementary material for: Role of Hepcidin in the Setting of Hypoferremia during Acute Inflammation
Source: PLoS One. 2013 Apr 23;8(4):e61050. doi: 10.1371/journal.pone.0061050 (PMC3634066; doi:10.1371/journal.pone.0061050)
Supplement: Table S1 — Primers sequences used for Real-time PCR. (DOCX) [file pone.0061050.s006.docx]

Table S1: Primers sequences used for Real-time PCR

| Gene name | Primer | Sequence (5’–3’) |
| --- | --- | --- |
| *Hepcidin1* | Forward | CCT ATC TCC ATC AAC AGA T |
|  | Reverse | TGC AAC AGA TAC CAC ACT G |
| *Ferroportin* | Forward | TTG CAG GAG TCA TTG CTG CTA |
|  | Reverse | TGG AGT TCT GCA CAC CAT TGA T |
| *Ferroportin 1A* | Forward | CCC ATA AGG CTT TGG CTT TCC A |
|  | Reverse | GTG GAG AGA GAG TGG CCA AG |
| *Ferroportin 1B* | Forward | AAG CCG GTT GGA GTT TCA ATG TT |
|  | Reverse | GTG GAG AGA GAG TGG CCA AG |
| *Il6* | Forward | CTT CAA CCA AGA GGT AAA AGA TTT A |
|  | Reverse | TAG GAG AGC ATT GGA AAT TGG GGT AGG AAG G |
| *TNF-α* | Forward | GAA CTG GCA GAA GAG GCA CT |
|  | Reverse | GGT CTG GGC CAT AGA ACT GA |
| *Heme Oxygenase1* | Forward | GTC AAG CAC AGG GTG ACA GA |
|  | Reverse | ATC ACC TGC AGC TCC TCA AA |
| *Activin B* | Forward | TCA GCT TTG CAG AGA CAG |
|  | Reverse | GAA GAA GTA CAG GCG GAC |
| *Lipocalin-2* | Forward | GAG CTA CAA TGT GCA AGT GG |
|  | Reverse | CAG AGA AGA TGA TGT TGT CG |
| *Ferritin L* | Forward | GGG CCT CCT ACA CCT ACC TC |
|  | Reverse | CTC CTG GGT TTT ACC CCA TT |
| *Ferritin H* | Forward | GAC CGA GAT GAT GTG GCT CT |
|  | Reverse | GTG CAC ACT CCA TTG CAT TC |
| *Tfr1* | Forward | TCC GCT CGT GGA GAC TAC TT |
|  | Reverse | TCA AGT TCT CCA CTA AAG C |
| *Tfr2* | Forward | GGT CCT GAT CAC CCT GCT AA |
|  | Reverse | GGA GGT CGC TCC AGT ACA AC |
| *BMP6* | Forward | GTT CCG CGT CTA CAA GGA CT |
|  | Reverse | CAG CCA ACC TTC TTC TGA GG |
| *Matriptase-2* | Forward | CCT GGT GAG TTC CTC TGC TC |
|  | Reverse | CTT GGC ACT GTT CTT CGT CA |
| *HFE* | Forward | GTC TGT GCC ATC TTC TTG GTT GG |
|  | Reverse | CTA CAG TGG AGC AAG TGT GCC |
| *Hemojuvelin* | Forward | CCC AGA TCC CTG TGA CTA TGA |
|  | Reverse | CAG GAA GAT TGT CCA CCT CAG |
| *Alk3* | Forward | AAG TTG CTG TAT TGC TGA CC |
|  | Reverse | CTT CTG GAG CCA TGT ACC GC |
| *Neogenin* | Forward | CCC TGG TCT CTA CTC GCT TC |
|  | Reverse | CCT GGC TGG CTG GTA TTC TC |
| *Smad6* | Forward | GTT GCA ACC CCT ACC ACT TC |
|  | Reverse | GGA GGA GAC AGC CGA GAA TA |
| *Smad7* | Forward | GCA GGC TGT CCA GAT GCT GT |
|  | Reverse | GAT CCC CAG GCT CCA GAA GA |
| *Dcytb* | Forward | ACG GTT CTC ATG GGA GTG AC |
|  | Reverse | GAA GGC CCA GCG TAT TTG TA |
| *DMT1+IRE* | Forward | TGT TTG ATT GCA TTG GGT CTG |
|  | Reverse | CGC TCA GCA GGA CTT TCG AG |
| *Cyclophilin* | Forward | ATG GCA CTG GCG GCA GGT CC |
|  | Reverse | TTG CCA TTC CTG GAC CCA AA |
